# Successful coil embolization with distal radial access for a ruptured radial artery pseudoaneurysm in a patient with SARS‐CoV‐2 infection

**DOI:** 10.1002/ccr3.5509

**Published:** 2022-03-01

**Authors:** Soichiro Washimi, Takeshi Yamada, Akihiko Takahashi

**Affiliations:** ^1^ Department of Cardiology Sakurakai Takahashi Hospital Kobe Japan

**Keywords:** coil embolization, distal radial access, pseudoaneurysm

## Abstract

A 50‐year‐old man with a medical history of diabetes mellitus and hypertension was admitted to a university hospital for severe respiratory insufficiency caused by SARS‐CoV‐2 infection. His arterial blood pressure and blood oxygen levels were monitored through a plastic cannula inserted in the radial artery in the intensive care unit. After recovery from respiratory insufficiency, the patient was moved to a local hospital where hematoma formation and skin necrosis at the puncture site were noted. On the 25th day from the initial admission, the patient developed massive bleeding from the wound on the hematoma and was transferred to our hospital on emergency. A 6‐Fr sheath introducer was inserted through the right distal radial artery in the anatomical snuff box, and instant hemostasis was accomplished without external compression. Thereafter, percutaneous thrombin injection for the pseudoaneurysm was conducted under ultrasound guidance. However, bleeding from the pseudoaneurysm was still observed after radial sheath withdrawal. We then performed coil embolization of the radial artery, which involved a coil proximal to the aneurysm, four coils in the neck of the aneurysm, and two coils distal to the aneurysm. Permanent hemostasis was achieved with no further vascular complications.

## Graphical abstract

The feasibility of a percutaneous catheter intervention with coil embolization even for the emergency case with ruptured radial pseudoaneurysm, which previously has been considered to mandate surgical intervention, was presented.

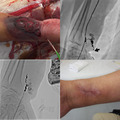

## 1 INTRODUCTION

Pseudoaneurysms of radial artery usually occur due to inadequate hemostasis after radial artery cannulation or catheterization. Usually, these patients are safely managed with ultrasound‐guided mechanical compression or thrombin injection; however, in the case of a ruptured pseudoaneurysm, this is complicated by active bleeding and skin necrosis associated with possible infection. To date, only a small number of rupture cases have been reported; surgical repair was conducted in all cases.^1^, ^2^, ^3^ In this study, we describe our experience of a patient with a large, ruptured radial pseudoaneurysm and SARS‐CoV‐2 infection that was treated with percutaneous intervention.

## 2 CASE REPORT

**FIGURE 1:**
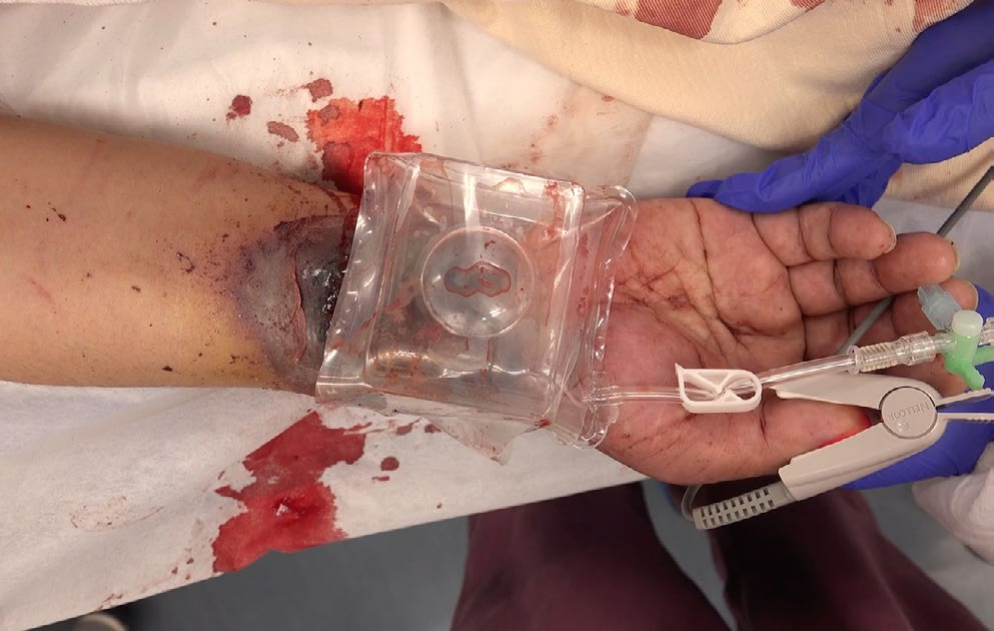
After applying hemostatic device on the bleeding site in the emergency room, hemostasis is temporally achieved

**FIGURE 2:**
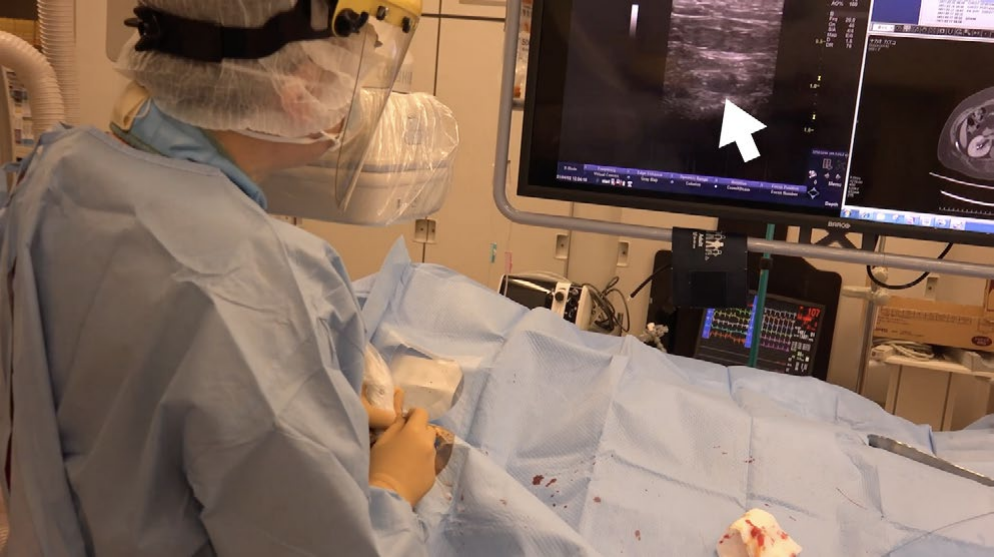
After local anesthesia is administered in the right snuff box, a 23 G plastic needle (white arrow) is used to successfully penetrate the distal radial artery under sonographic guidance

**FIGURE 3:**
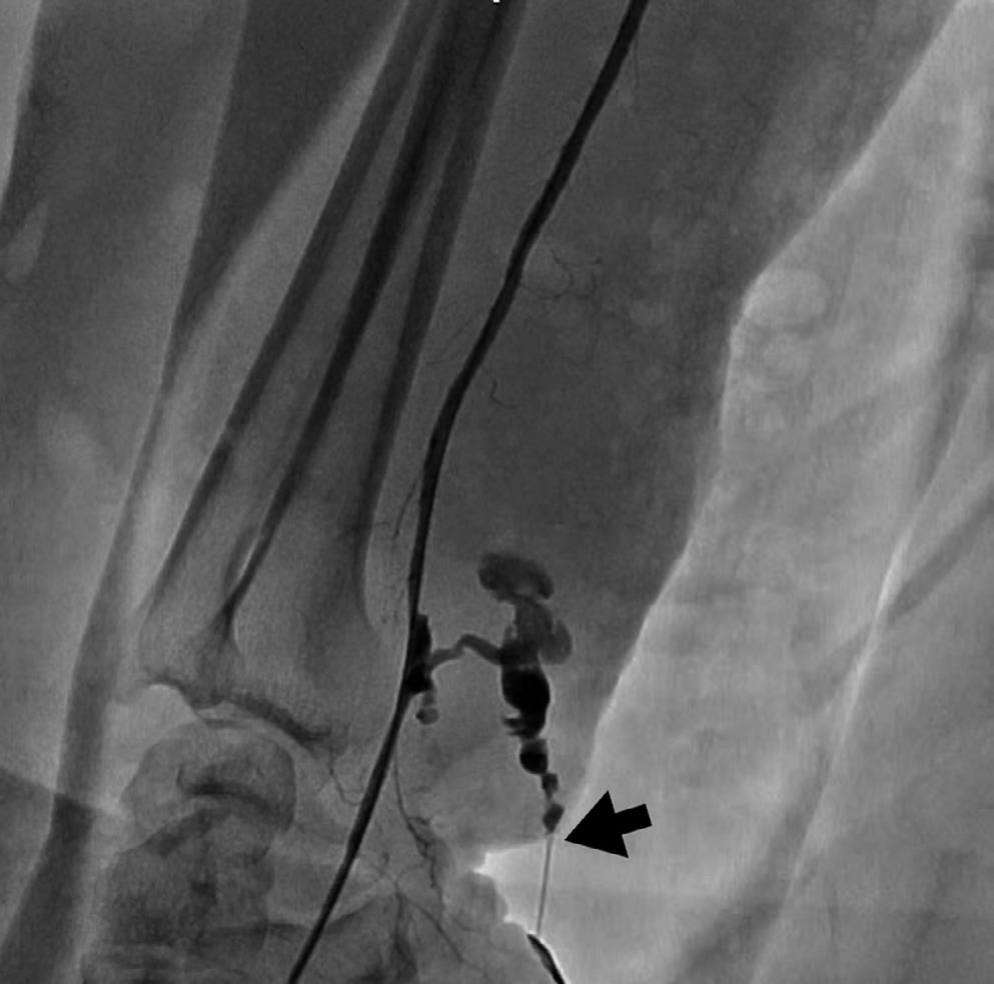
Radial artery angiography performed through the introducer showing laceration of the radial artery and active bleeding through the pseudoaneurysm (black arrow)

**FIGURE 4:**
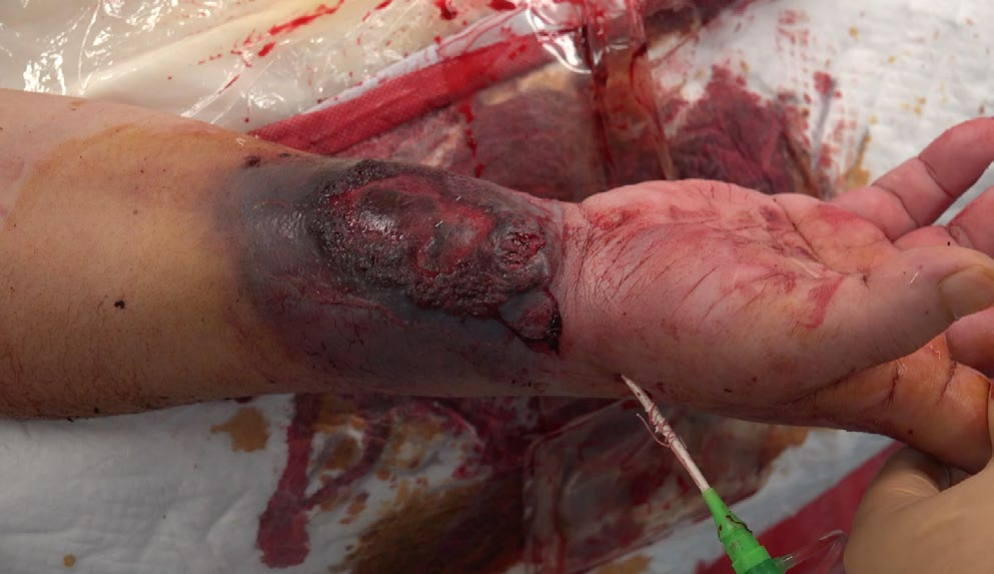
Hemostasis is completed without any external compression after a 6‐Fr sheath introducer of length 16 cm (6‐Fr Glidesheath™, Terumo) was fully inserted from distal radial artery

**FIGURE 5:**
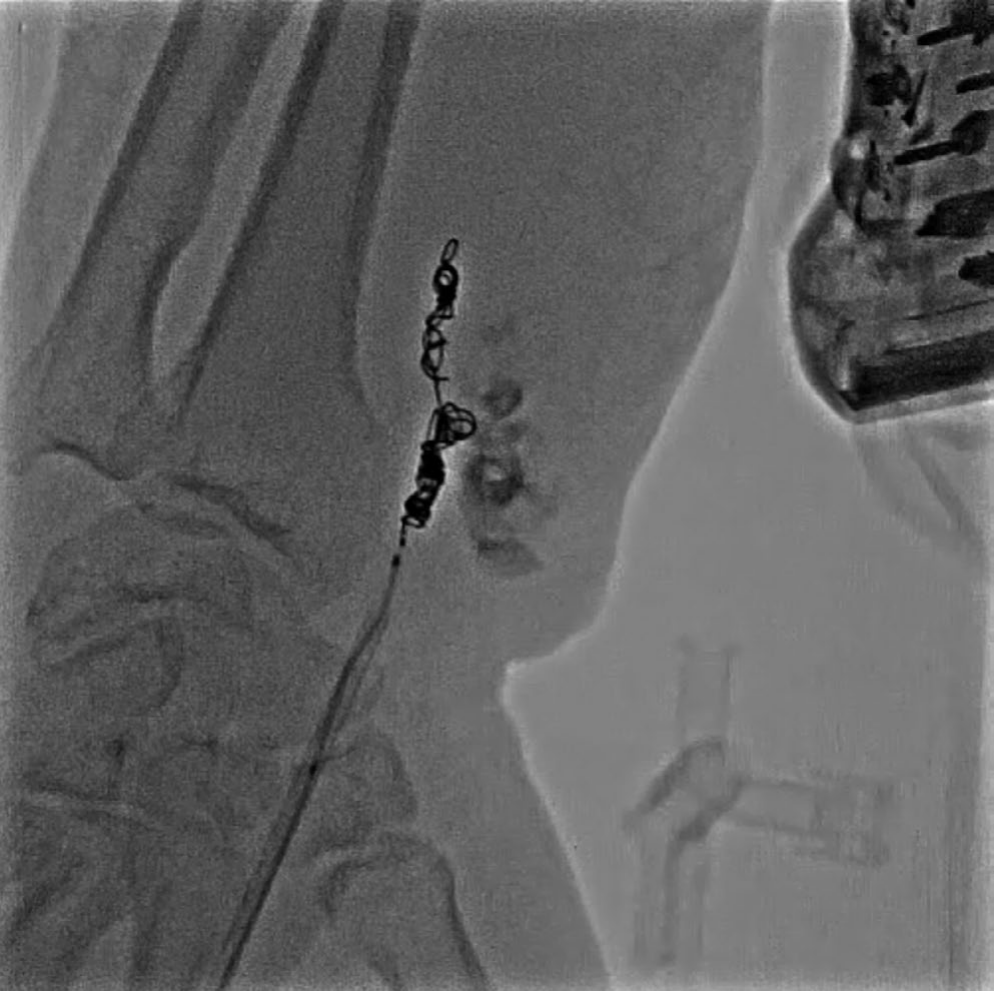
Fluoroscopy showing 4 coils implanted in the neck of the aneurysm. (Interlock™ Fibered IDC™ Occlusion System 4 × 80 mm, 4 × 150 mm, 4 × 150 mm, Boston Scientific™; Target XL Detachable Coils 3 × 90 mm Striker Corporation)

**FIGURE 6:**
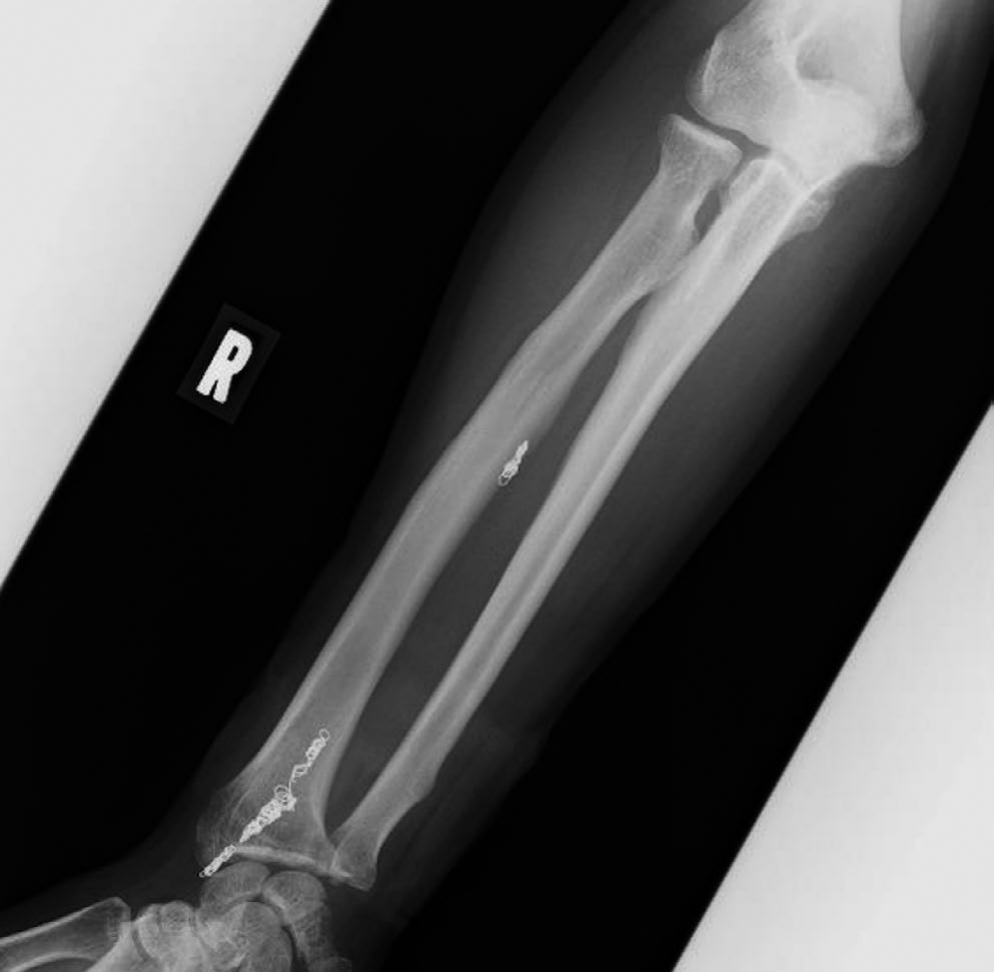
X‐ray photograph showing a coil (Interlock™ Fibered IDC™ Occlusion System 4 × 80 mm, Boston Scientific™) in the site proximal to the aneurysm, 4 coils in the neck of the aneurysm, (Interlock™ Fibered IDC™ Occlusion System 4 × 80 mm, 4 × 150 mm, 4 × 150 mm, Target XL Detachable Coils 3 × 90 mm Striker Corporation) and 2 coils distal to the aneurysm (Target XL Detachable Coils 2 × 60 mm, 2 × 60 mm were implanted). Hemostasis was achieved successfully after coiling of radial artery

**FIGURE 7:**
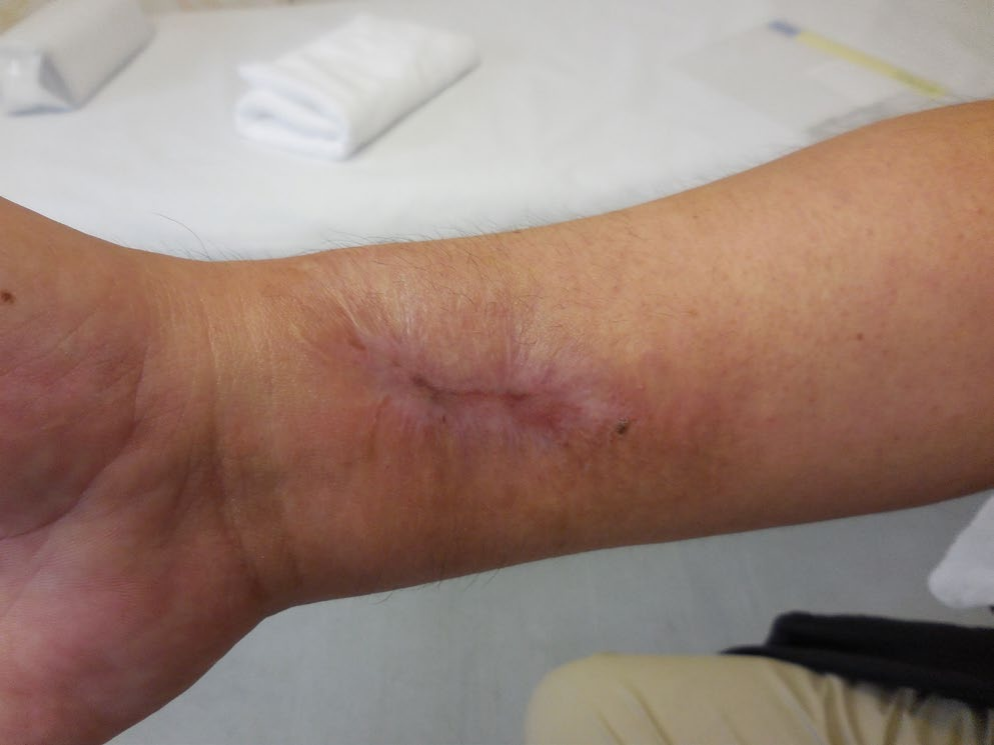
Photograph taken 6 months later showing that the wound had healed completely

A 50‐year‐old man, with a medical history of diabetes mellitus and hypertension, was admitted to the university hospital for acute respiratory insufficiency caused by SARS‐CoV‐2 infection. He was provided respiratory support with a high‐flow nasal cannula (HFNC) in the intensive care unit under combination therapy with dexamethasone, remdesivir, and tocilizumab. His arterial blood pressure and blood oxygen content were monitored through a plastic cannula inserted in the radial artery of the right forearm during HFNC support. Radial arterial cannulation was removed after his condition improved. Hematoma formation and skin necrosis at the puncture site were noted after the patient was moved to the local hospital for post‐SARS‐CoV‐2 infection care. However, the patient refused any treatment, including manual compression or surgical intervention. On the 25th day after initial admission, the patient developed massive bleeding from the wound on the hematoma. He was referred to our hospital for manual compression by a doctor. Hemostasis was temporarily achieved following the application of a pneumatic tourniquet on the upper arm and hemostatic device on the bleeding site in the emergency room (Figure 1). He was transferred to the catheter laboratory and underwent distal radial cannulation; after local anesthesia was administered in the right snuff box, a 23 G plastic needle was successfully inserted into the distal radial artery with sonographic guidance (Figure 2). A 0.025 silver guidewire (Asahi Intecc Co., Ltd.) was advanced to the brachial artery, and a 3‐Fr sheath introducer was inserted. Radial artery angiography performed through the introducer showed laceration of the radial artery and active bleeding (Figure 3). A 6‐Fr sheath introducer with a length of 16 cm (6‐Fr Glidesheath™, Terumo) was then fully inserted from the distal radial artery after retrieval of the 3‐Fr sheath introducer. Consequently, no bleeding was observed without external compression (Figure 4), and the sheath introducer was left in the radial artery for hemostatic purposes. The following day, 3000 units of percutaneous thrombin was administered under ultrasound guidance. However, bleeding from pseudoaneurysm was still observed after radial sheath withdrawal. We then performed coil embolization on the radial artery, which involved a coil (Interlock™ Fibered IDC™ Occlusion System 4 × 80 mm, Boston Scientific™) proximal to the aneurysm, four coils in the neck of the aneurysm, (Interlock™ Fibered IDC™ Occlusion System 4 × 80 mm, 4 × 150 mm, 4 × 150 mm, Target XL Detachable Coils 3 × 90 mm Striker Corporation) and two coils distal to the aneurysm (Target XL Detachable Coils 2 × 60 mm, 2 × 60 mm). Consequently, hemostasis was achieved successfully after coiling of the radial artery (Figures 5 and 6). The cultures taken from the wound and arterial blood showed negative results. Twenty‐five days after the procedure, the patient was transferred to the previous hospital for further treatment. The wound healed completely 3 months after discharge (Figure 7).

## 3 DISCUSSION

Placing a plastic cannula in the radial artery to monitor arterial blood pressure and access samples for blood gas testing^4^ is considered safe, with a vascular complication rate <0.5%.^5^ Furthermore, the incidence of pseudoaneurysm after radial artery catheterization is low, occurring in 0.09% of cases.^6^ Various methods have been proposed to treat pseudoaneurysms, including simple compression with an echo probe, percutaneous thrombin injection, and prolonged placement of a sheath introducer.^7^ However, only a limited number of ruptured pseudoaneurysm cases treated with a surgical approach have been reported.^1^, ^2^, ^3^ This is the first reported case to have been treated with a percutaneous intervention for the ruptured radial pseudoaneurysm using a distal radial approach.

Distal radial access has been used as an alternative for conventional radial access during coronary catheterization and intervention because of its possible advantages correlated with vascular complications and patient comfort.^8^ Distal radial access also can be considered as a suitable access site to treat, as it is located on the distal side of the conventional radial access.^9^ In the current case, we achieved instant hemostasis by placing the 6‐Fr sheath introducer in the radial artery from the distal radial artery in the snuff box. Since we could not achieve permanent hemostasis with thrombin injection into the lumen of the pseudoaneurysm, we conducted a hemostatic coil embolization in the radial artery from the distal radial artery.

Antegrade approach including brachial access or ipsilateral ulnar access^10^ was also considered applicable for the treatment of radial aneurysm. Antegrade access has advantages such as flexibility in positioning of the coils in the distal part of aneurysm and reliable angiographic effect, compared to that of distal radial access described above; therefore, it may be useful in certain cases.

Several reports have suggested that surgical vascular reconstruction should be considered to maintain the blood supply to the hand by the radial artery.^10^, ^11^ Further, a more recent report suggested that vascular intervention using distal radial access with a covered stent was appropriate for non‐ruptured radial pseudoaneurysms.^12^ In the current case, however, the patient's reduced immune status caused by diabetes mellitus and the administration of immunosuppressive drugs, such as dexamethasone and tocilizumab, needed for the treatment of COVID‐19 pneumonia, may have contributed to a possible local infection, even though the cultures of the wound and arterial blood were negative. Further, the patient still refused any surgical intervention. Therefore, we avoided vascular reconstruction with a covered stent or surgical revascularization with autologous saphenous vein.

We believe that the current hemostatic technique is a possible alternative to the known methods for the treatment of ruptured pseudoaneurysms in the radial artery, in particular, for cases where wound infection is highly suspected.

## 4 CONCLUSION

We report our experience of treating a ruptured radial pseudoaneurysm using coil embolization from the distal radial artery after failed insertion of an arterial sheath and thrombin injection. This method can achieve instant hemostasis and is considered a safe approach, even in cases where wound infection is suspected.

## CONFLICTS OF INTEREST

The authors declare no conflicts of interest.

## AUTHOR CONTRIBUTIONS

S.W and T.Y were involved in data preparation and data interpretation. A.T was involved in the draft writing. All authors critically revised the report, commented on drafts of the manuscript, and approved the final report.

## ETHICAL APPROVAL

This study was performed in accordance with the Helsinki Declaration and ethics approvals were obtained from the Institutional Review Boards of the authors' hospital.

## CONSENT

Written informed consent was obtained from the patient to publish this report in accordance with the journal's patient consent policy.

## Data Availability

Data sharing not applicable to this article as no datasets were generated or analysed during the current study.
